# Quality indicators for Palliative Day Services: A modified Delphi study

**DOI:** 10.1177/0269216318810601

**Published:** 2018-11-19

**Authors:** Noleen K McCorry, Sean O’Connor, Kathleen Leemans, Joanna Coast, Michael Donnelly, Anne Finucane, Louise Jones, W. George Kernohan, Paul Perkins, Martin Dempster

**Affiliations:** 1 Centre of Excellence for Public Health, Centre for Public Health, Queen’s University Belfast, Belfast, UK; 2 School of Nursing, Ulster University, Newtownabbey, UK; 3 End-of-Life Care Research Group, Vrije Universiteit Brussel, Brussels, Belgium; 4 Department of Radiotherapy and Supportive Care, Universitair Ziekenhuis Brussel, Vrije Universiteit Brussel, Brussels, Belgium; 5 Health Economics at Bristol, Population Health Sciences, Bristol Medical School, University of Bristol, Bristol, UK; 6 Marie Curie Hospice, Edinburgh, UK; 7 Usher Institute of Population Health Sciences and Informatics, University of Edinburgh; 8 University College London, London, UK; 9 Sue Ryder Leckhampton Court Hospice, Cheltenham, UK; 10 Gloucestershire Hospitals NHS Foundation Trust, Cheltenham, UK; 11 School of Psychology, Queen’s University Belfast, Belfast, UK

**Keywords:** Palliative care, Delphi technique, quality indicators, healthcare, day services, medical, quality improvement

## Abstract

**Background:**

The goal of Palliative Day Services is to provide holistic care that contributes to the quality of life of people with life-threatening illness and their families. Quality indicators provide a means by which to describe, monitor and evaluate the quality of Palliative Day Services provision and act as a starting point for quality improvement. However, currently, there are no published quality indicators for Palliative Day Services.

**Aim:**

To develop and provide the first set of quality indicators that describe and evaluate the quality of Palliative Day Services.

**Design and setting:**

A modified Delphi technique was used to combine best available research evidence derived from a systematic scoping review with multidisciplinary expert appraisal of the appropriateness and feasibility of candidate indicators. The resulting indicators were compiled into ‘toolkit’ and tested in five UK Palliative Day Service settings.

**Results:**

A panel of experts independently reviewed evidence summaries for 182 candidate indicators and provided ratings on appropriateness, followed by a panel discussion and further independent ratings of appropriateness, feasibility and necessity. This exercise resulted in the identification of 30 indicators which were used in practice testing. The final indicator set comprised 7 structural indicators, 21 process indicators and 2 outcome indicators.

**Conclusion:**

The indicators fulfil a previously unmet need among Palliative Day Service providers by delivering an appropriate and feasible means to assess, review, and communicate the quality of care, and to identify areas for quality improvement.

**What is already known about the topic?**

- Measurement of healthcare quality creates the basis for quality improvement.
- Quality indicators can provide a valid and reliable means of measuring quality of care.
- There are currently no published quality indicators specifically for Palliative Day Services.

**What this paper adds?**

- This paper describes the development of the first set of quality indicators specifically for quality improvement in Palliative Day Services.
- The final set comprises 7 structural indicators (e.g. ‘Service has a written standard operating procedure for development and use of multidisciplinary care plans’), 21 process indicators (e.g. ‘Proportion of service users with assessment of pain severity at screening using a valid measure’) and 2 outcome indicators (e.g. ‘Proportion of service users re-assessed at regular review who report that main care goals are met’).

**Implications for practice, theory or policy**

- The quality indicator set offers day service providers with a means of describing and reviewing the quality of their care, and providing feedback to stakeholders.
- Use of the indicator set in practice will allow providers to identify areas for quality improvement.

## Introduction

Quality indicators are statements that define explicitly and in measurable terms the quality of a given construct or phenomenon. They provide a means with which to describe, monitor and evaluate healthcare.^1^ Ideally, they should be evidence-based with a theoretical foundation such as Donabedian’s^2^ structure, processes and outcomes framework. Quality indicators can provide service users, their families, care staff, providers, commissioners, purchasers and inspectorates of care with data in relation to the quality of care, sometimes against benchmarks or previous quality assessments. In addition, by providing a valid and reliable means of measuring quality of care, quality indicators (although not sufficient by themselves) can act as a starting point for quality improvement.^3^

In the United Kingdom, as in many European countries, Palliative Day Services provide specialist palliative care within a group context for people with terminal or life-limiting illness, facilitated by a specialist multidisciplinary team.^4^ The goal of Palliative Day Services is to provide individualised, holistic care that promotes independence and rehabilitation, improves self-worth and ultimately enables the best quality of life for patients and their families.^4,5^ However, there is considerable variation within Palliative Day Services, and providers are under pressure to define and measure the quality of their services, identify areas for improvement and assess the impact of service development and improvement efforts. In order to address these issues, we developed the first set of quality indicators that are designed specifically for use by Palliative Day Services. We propose that our indicators be used to support services to evaluate care quality on an ongoing basis, to identify valid and appropriate goals for quality improvement.

## Methods and results

**Figure 1.**
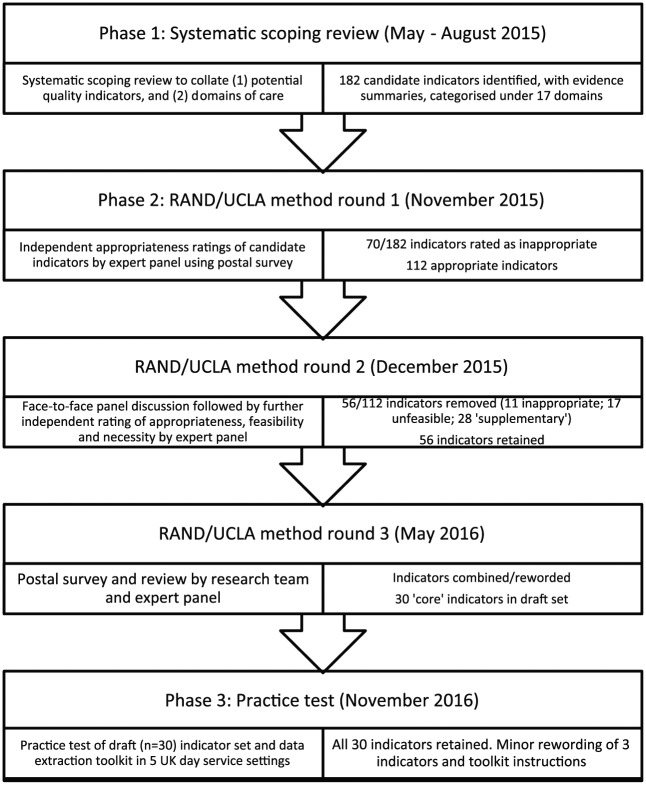
Phases in the research process, including number of potential quality indicators identified at each stage.

We used the Research ANd Development/University of California, Los Angeles (RAND/UCLA) appropriateness method^6^ which has been incorporated into a comprehensive approach for the development of quality indicators in palliative care.^7^ The RAND/UCLA appropriateness method (RAM) is a modified Delphi method which combines the use of evidence with the collective judgement of experts and is particularly suited to this area of healthcare because of the dearth of evidence related to day services. Expert panellists provide two rounds of *independent* ratings and have the opportunity to discuss their judgements between the rating rounds during a face-to-face meeting. The method has been shown to have a high level of reproducibility and validity.^6^ Figure 1 shows the phases in the research process.

The study protocol was approved by the Research Ethics Committee, School of Psychology, Queen’s University Belfast (ref: 10-2015-16) in September 2015. Expert panel members provided written informed consent to participate in the study.

### Phase 1 - a systematic scoping review to identify existing quality indicators and domains

A systematic scoping review was conducted to identify existing quality indicators in all areas of palliative care and other evidence or recommendations which might inform the development of (or translation of evidence into) a quality indicator, that is, structural or process-level variables which have been shown to be related to the outcomes of care. Any domains/themes used to describe the indicators were also identified. Established frameworks were used to guide the review protocol,^8,9^ which is published elsewhere.^10^

This review resulted in the identification of 182 unique candidate indicators (supplementary file 1) and 17 care domains. Evidence tables summarising the content, sources and quality of evidence (using AIRE (Assessment of Indicators through Research and Evaluation) Instrument^11^ and GRADE (Grading of Recommendations, Assessment, Development and Evaluations)^12^ scores where appropriate) of quality indicators represented in each domain were compiled.

### Phase 2 - quality indicator selection by expert consultation following the internationally validated RAM

#### Round 1 - expert panel questionnaire

A multidisciplinary panel of individuals with expertise in Palliative Day Services was established. A total of 58 potential panel members were directly approached (recommended by the research team) or responded to advertisements. We selected experts based on their experience and expertise in the area of Palliative Day Services, while aiming for diversity in geographical representation and professional specialism. Panel members were asked to commit 21 h to the project over 3 months. The resultant panel consisted of 21 individuals from across the United Kingdom, including palliative care consultants, specialist nurses, day service and hospice managers, allied health professionals, spiritual care providers, a social worker, a complementary therapist, a psychologist and a pharmacist. Several panellists had experience of more than 1 day of service model and previous experience of consensus development methods. Panel members were sent a document pack including general information about quality indicators and their properties (including a description of Donabedian’s^2^ model), a series of evidence tables for candidate quality indicators, a rating booklet and a short commentary from Marie Curie (a major UK palliative care provider) endorsing the project objectives. All materials were reviewed by the research team and two user representatives.

Panellists were asked to independently rate the *appropriateness* of each quality indicator on a 9-point scale (according to the RAM^6^) where an appropriate indicator (rated 7–9) was defined as one which was acceptable and likely to represent a reasonable measure of quality in Palliative Day Services. Alternatively, an inappropriate indicator (rated 1–3) was defined as one which should rarely or never be used as a measure of quality in Palliative Day Services, and where any re-wording or reframing of the indicator would not alter this assessment. Ratings of 4–6 represented indicators which were thought to be neither appropriate nor inappropriate. Panellists were asked to base ratings on their own knowledge and experience, as well as the evidence summary tables provided, but not to rate appropriateness based on the cost implications associated with a particular indicator. It was explained to panellists that although cost consideration is an important factor in deciding whether a particular procedure or treatment is ultimately made available to patients, the RAM focuses only on the initial question of whether it is effective. Panellists were also asked to suggest additional quality indicators, and to review and provide feedback on the proposed quality domains. The indicator ratings were subsequently analysed based on their median appropriateness scores and the level of agreement between panel members using the criteria specified by the RAM.^6^ Indicators with a median appropriateness rating of 3 or less (inappropriate) and good agreement on this rating by the panel were highlighted for exclusion.

Twenty panellists returned round 1 ratings. Round 1 resulted in the identification of 70 inappropriate indicators and 112 candidate appropriate or uncertain indicators.

#### Round 2 - expert panel meeting

All panellists who participated in round 1 were invited to attend a 1-day, face-to-face meeting, where the analysis of round 1 ratings was presented. The meeting was moderated by a health psychologist (M.De.) with extensive experience in facilitating group discussion and was attended by 12 panellists. The aims of the meeting were as follows:

1. Confirm the exclusion of indicators rated as inappropriate as a result of round 1 ratings;
2. Discuss those indicators for which, following round 1, appropriateness was uncertain;
3. Discuss indicators for which appropriateness was acceptable but there was disagreement between panellists;
4. Review the terminology used in indicator descriptions.

Following the discussions, panellists were asked to independently re-rate the *appropriateness* of the 112 indicators. Panel members agreed that the indicator set should be designed and promoted as a tool to support the assessment of quality in a formative manner, and the identification of quality improvement goals, and not as a means of comparison between services or for inspection purposes – which would require more detailed consideration of risk adjustments. At this stage, panel members were also asked to independently rate the *feasibility* of measuring each indicator in the day service setting using the same 9-point scale and to assess if each indicator was a *necessary* measure (yes/no response). A necessary measure was defined as follows: *appropriate; likely to benefit the patient; that the benefit is not small*; and *where it would be improper care not to offer the procedure under review*.^6^ The same criteria as round 1 were used to remove inappropriate indicators. In addition, only quality indicators with a median feasibility rating of 4 or greater (with good agreement) were retained. The categorisation of necessity was used to produce a list of core and supplementary indicators. Indicators were defined as supplementary if less than half the panel identified the indicators as a necessary measure.

As a result of round 2 ratings, 11 indicators were agreed to be inappropriate and 17 were unfeasible. A further 28 indicators were removed based on the assessment of necessity. Hence, 56 core indicators (agreed to be appropriate, feasible and necessary) were retained following round 2. Supplementary file 1 shows the decisions during rounds 1 and 2.

#### Round 3 - second panel questionnaire

The set of 56 core indicators was then reviewed by the research team and expert panel members (with a particular focus on wording and duplication) who were sent the indicator set by email.

Round 3 resulted in the re-wording or combination of 41 indicators, and hence a consolidated set of 30 unique indicators. Supplementary file 2 shows the derivation of the draft indicator set from the original 182 candidate indicators. This draft indicator set included 7 structural indicators, 21 process indicators and 2 outcome indicators, categorised under 10 domains of care. There were most quality indicators (*n* = 9) representing the domain ‘co-ordination and continuity of care’. For 24 of the indicators, the focus is on patient or staff interaction with the service (e.g. ‘Proportion of service users with assessment of pain severity at screening using a valid measure’ (#A1)), while 6 indicators represented service characteristics (e.g. ‘Service has a written care pathway for assessment and management of moderate or severe pain including appropriate onward referral routes’ (#E12)).

### Phase 3 - testing the draft quality indicator set in practice

The draft indicator set was compiled into a toolkit with detailed descriptions of each quality indicator (including the numerator, denominator and definitions) and instructions to assist with the extraction of relevant data. Supplementary file 3 is an extract from the toolkit. The indicator set and toolkit were then field tested in five UK Palliative Day Service settings, in England (2), Scotland (1) and Northern Ireland (2) representing three different palliative care providers. The toolkit instructed data abstractors to assess performance on each quality indicator using paper-based or electronic records for 15 consecutive patients discharged from the Palliative Day Service in the previous 12 months (for patient-level indicators) and any relevant accessible documentation including service policies and procedures (for service-level indicators). Day service managers at each site completed the data abstraction. Abstractors were asked to ‘think aloud’^13^ while completing the paperwork – so that challenges or misunderstandings could be readily identified by the researcher, who was available during the entire abstraction process.

In total, data were extracted from 82 patient records. Following completion of the practice test, the rate and variation in the proportion of patients/staff for whom each quality indicator was met and the proportion of settings which satisfied the service-level indicators were compiled (supplementary file 4). Overall, there was considerable variation across the five services in performance against the indicators, particularly for assessment of patient satisfaction, recording of care goals and completion of care plans. The indicators that were least likely to be met were concerned with quality-of-life assessment, availability of a completed multidisciplinary care plan and assessment of patient satisfaction with support for decision-making, with some services not collecting any information on patient satisfaction or quality of life. The indicators that were most likely to be met were concerned with the documentation of time from referral to first attendance date offered; informed consent to treatment or medical intervention; and communication between the service and the general practitioner providing information on care needs and care plans. Feedback from data abstractors indicated the following:

- Data abstraction was perceived as time-consuming;
- Abstractors had to refer to several different sources of information;
- The paper-based extraction forms added to the cumbersome nature of the process;
- Data abstractors were not confident about the process for *calculation* of each indicator.

As a result of the practice test, minor amendments were made to three of the indicators and to the toolkit instructions. Table 1 shows the final (QualPalUK) quality indicator set.

**Table 1.** Final QualPalUK quality indicator set (n = 30), following phase 3 of the RAND/UCLA appropriateness method.

	Indicator description, categorised by care domain	Indicator type
A	*Physical care and support, assessment and treatment*	
A1	Proportion of service users with assessment of pain severity at screening using a valid measure	P
A2	Proportion of service users with moderate or severe pain assessed to explore possible causes of pain	P
A3	Proportion of service users with assessment of breathlessness at screening using a valid measure	P
A4	Proportion of service users with assessment of fatigue at screening using a valid measure	P
A5	Proportion of service users with assessment of functional status to identify daily activity limitations completed before a multidisciplinary care plan	P
B	*Psychological care and support, assessment and treatment*	
B6	Proportion of service users screened for depression at screening using a valid measure	P
B7	Proportion of service users screened for anxiety at screening using a valid measure	P
B8	Proportion of service users with assessment of cognitive functioning	P
C	*Spiritual and emotional care and support*	
C9	Proportion of service users with documentation of a ‘spiritual aspects of care discussion or assessment’ completed before a multidisciplinary care plan	P
D	*Information and communication with service users*	
D10	Proportion of service users who report that they are provided with sufficient, appropriately tailored information or advice on their condition and on intervention options to support decisions on agreed care planning	O
E	*Co-ordination and continuity of care*	
E11	Proportion of service users with a comprehensive needs assessment completed before a multidisciplinary care plan to identify main symptoms and concerns, and their effect	P
E12	Service has a written care pathway for assessment and management of moderate or severe pain including appropriate onward referral routes	S
E13	Proportion of service users with documentation of re-assessment at regular review in line with time points agreed in the multidisciplinary care plan	P
E14	Service has written standard operating procedures defining timeframes for time to initial contact, completion of needs assessment and multidisciplinary care plan	S
E15	Proportion of service users with documentation of appropriate intervention in line with the agreed, multidisciplinary care plan	P
E16	Proportion of service users with documented communication between the service and the general practitioner providing information on care needs and care plans	P
E17	Proportion of service users with a care plan available as specified by the service’s written standard operating procedure for development and usage of multidisciplinary care plans	P
E18	Proportion of service users with documented evidence of being offered the opportunity for completion of advance care planning	P
E19	Proportion of service users with quality of life assessed using a valid measure at screening and at regular review in line with time points agreed in the multidisciplinary care plan	P
F	*Care planning, goal setting and shared decision-making with service users*	
F20	Service has a written standard operating procedure for development and use of multidisciplinary care plans	S
F21	Proportion of service users with documentation of main care goals in the multidisciplinary care plan	P
G	*Evidence of effectiveness, outcome assessment and measurement*	
G22	Service has a written policy for reviewing and updating standard operating procedures and care pathways	S
G23	Proportion of service users re-assessed at regular review who report that main care goals are met in line with the multidisciplinary care plan	O
G24	Proportion of service users with assessment of satisfaction with overall care and support performed using a valid measure	P
G25	Proportion of service users with assessment of satisfaction with involvement in shared decision-making	P
H	*Staff training and education, service and professional development*	
H26	Extent to which staff have access to training around core components of care as part of continuing education and personal development	S
I	*Access to services and service environment*	
I27	Proportion of service users with a record of time in days from referral date to first attendance date offered by service	P
I28	The service provides suitable equipment and settings to deliver care	S
I29	Service has a written policy for defining standards for equipment and settings which are available for delivery of care	S
J	*Societal, ethical and legal aspects of care*	
J30	Proportion of service users with correctly completed documentation of informed consent to treatment or medical intervention	P

RAND/UCLA: Research ANd Development/University of California, Los Angeles; S: structure; P: process; O: outcome.

## Discussion

### Results of the study

We have developed the first set of quality indicators specifically for use in Palliative Day Services, using a recommended, evidence-based approach.^7^ The indicators were derived from a comprehensive review of the international literature. The full set of original 182 indicators is provided as a resource in supplementary material and can be used to make adjustments for jurisdictions outside the United Kingdom if necessary. The final indicator set (reflecting Donabedian’s^2^ model) contains 2 outcome, 21 process and 7 structural indicators, across 10 domains of care. The limited number of outcome indicators is a result of the expert panel’s preference to incorporate patient-reported outcome measurement (in relation to the assessment of pain, breathlessness, fatigue, functional status, depression, anxiety and quality of life) into relevant *process* and s*tructural* indicators, and to avoid the complex adjustment and exclusions often associated with the quality appraisal using *outcome* indicators.^14–19^ For example, rather than measure absolute ‘pain intensity’ or ‘change in pain intensity’ (both outcome indicators), the panel preferred to measure the extent to which patients had their pain measured using a validated instrument (#A1 and #A2 - both process indicators) and the extent to which valid pathways were in place to manage the individual patient’s pain (#E12 - a structural indicator). This approach still incorporates the perspective of the service user in the process of quality assessment,^20,21^ but requires that staff solicit these patient-reported outcomes routinely and use them effectively to meet patient needs. Clearly, however, both structural and process-level quality indicators are only valid assessments of quality of care if they can be shown to increase the likelihood of a good outcome,^22^ and hence, the evidence base should be reviewed regularly to identify these relationships. The panel did, however, endorse *outcome* indicators in relation to service users’ satisfaction with information and advice received (#D10) and whether service users reported that their main care goals had been met (#G23).

One characteristic of a ‘good quality indicator’ is the extent to which the quality indicator refers to an aspect of care which can be influenced by the players being evaluated.^23^ Many quality indicators developed more recently^7,24–26^ have been proposed to be relevant to a range of different palliative care services. It is inevitable though that some of the indicators within these sets will not be within the control of those care personnel associated with the service being evaluated. Several authors have commented on this ‘fit’ between the indicator set and the service being evaluated^27,28^ and have recommended that indicators be amended or removed as appropriate. We believe that the specificity of our indicator set is a significant advantage as it means the indicator set is immediately accessible to UK Palliative Day Services, without modification. There is considerable scope for international collaboration in the development of quality indicators,^29,30^ and hence, with appropriate modifications to account for contextual and cultural differences, our indicator set will be valuable in other Palliative Day Services, internationally. The original set of 182 unique Palliative Day Services quality indicators (supplementary file 2) derived from a comprehensive review of the *international* literature is a valuable reference for other providers wishing to develop Palliative Day Services quality indicators.

### Implementation in practice

The value of quality indicators is fully realised when they are implemented in routine practice and used as a basis for quality improvement. Fifteen years after the Council of Europe first encouraged the definition and adoption of quality indicators of good palliative care, there is still little evidence of widespread implementation in practice.^16,31,32^ Some of the barriers to successful implementation of quality indicators in palliative care settings include the attitudes towards quality improvement within the organisation^27^ and among staff,^28^ the perceived value of quality indicators^27,28^ and ‘top-down’ engagement.^27^ Drawing upon this evidence and the improvement science literature,^33^ we have incorporated features in our research design which are intended to improve the likelihood of uptake and implementation by Palliative Day Services. Use of the rigorous RAM results in a set of indicators with high face and content validity.^34–36^ We have enhanced the perceived acceptability and credibility of the indicator set by promoting stakeholder awareness and involvement in the development of the indicators, and by ensuring representation on our expert panel from services where we wish the indicators to be utilised. We have communicated widely (via newsletters, presentations, the QualPalUK website and site visits) about the development process and have provided opportunities for stakeholder feedback. However, for successful implementation, we will also need to be attuned to variations in current practice, the range of measures already in place in care settings, the diversity of systems (including IT systems) and staff training.^28,37^

Assessment of care quality is agreed to be an essential element of service provision, and the quality indicator set is a comprehensive and evidence-based tool that enables this process. This comprehensive assessment requires time investment by services that are often time-poor, on an annual or bi-annual basis. Implementation will be facilitated where services are able to organise their routinely collected data in a manner that is easily accessible for data extractors; service personnel recognise the direct impact of quality assessment on service improvements; data extractors become more familiar with use of the tool; and where efficiency of data extraction is enhanced via electronic capture. We are now developing an electronic version of the quality indicator toolkit which will help to reduce the time required for data extraction and calculation of the quality indicators by allowing abstractors to input the required (prompted) fields, with calculations completed by the programme in the background. We will supplement the quality indicator toolkit with a quality improvement module which will support Palliative Day Services to first identify areas for quality improvement and subsequently to use Plan-Do-Study-Act cycles^38^ to work towards improvement.

Although there were only five practice sites, the practice test indicated that the assessment of satisfaction and quality of life, and the production (and communication of) comprehensive care plans and needs assessments are areas which may require attention within Palliative Day Services. This finding is consistent with existing literature which has demonstrated that, despite initiatives promoting the routine measurement of patient-reported outcomes^39–42^ and strong evidence of a positive effect on a multitude of care outcomes (including patient-clinician communication, patient satisfaction and identification of unrecognised symptoms),^43^ they are not yet widely measured in palliative care practice. Failure to implement patient-reported outcome measurement in palliative care has been attributed to barriers including fear of change, time management/ constraints, lack of education on use of tools, burden of tools for staff and service users, illness severity, concerns about criticism and cost constraints.^44^ The assessment of patient-reported outcomes is therefore one area where quality improvement projects may be particularly productive and valued by the Palliative Day Services community. In contrast, indicators which utilise administrative data that map onto the requirements of national^45^ or internal organisational audits were more likely to be met, such as ‘time from referral to first attendance’ or ‘consent to treatment’.

### Strengths and weaknesses

The RAM has been shown to produce indicators with high content^34,46^ and predictive validity.^32,47,48^ However, these characteristics and others (including sensitivity to change and reliability) should be field tested in a larger representative sample of Palliative Day Services, using the electronic toolkit for data extraction. In addition, the time commitment required from Delphi panellists often results in a panel that is atypical with respect to their interest and commitment to the topic being investigated. Generating interest in the value and implementation of Palliative Day Services quality indicators more widely is likely to be challenging.

### What this study adds

Our quality indicator set fulfils a need within the Palliative Day Services community, by providing a means of assessing and reviewing quality of care, and identifying areas for improvement.

## Supplemental Material

Supplementary_file_1 – Supplemental material for Quality indicators for Palliative Day Services: A modified Delphi study Click here for additional data file. Supplemental material, Supplementary_file_1 for Quality indicators for Palliative Day Services: A modified Delphi study by Noleen K McCorry, Sean O’Connor, Kathleen Leemans, Joanna Coast, Michael Donnelly, Anne Finucane, Louise Jones, W. George Kernohan, Paul Perkins and Martin Dempster in Palliative Medicine

## Supplemental Material

Supplementary_file_2 – Supplemental material for Quality indicators for Palliative Day Services: A modified Delphi study Click here for additional data file. Supplemental material, Supplementary_file_2 for Quality indicators for Palliative Day Services: A modified Delphi study by Noleen K McCorry, Sean O’Connor, Kathleen Leemans, Joanna Coast, Michael Donnelly, Anne Finucane, Louise Jones, W. George Kernohan, Paul Perkins and Martin Dempster in Palliative Medicine

## Supplemental Material

Supplementary_file_3 – Supplemental material for Quality indicators for Palliative Day Services: A modified Delphi study Click here for additional data file. Supplemental material, Supplementary_file_3 for Quality indicators for Palliative Day Services: A modified Delphi study by Noleen K McCorry, Sean O’Connor, Kathleen Leemans, Joanna Coast, Michael Donnelly, Anne Finucane, Louise Jones, W. George Kernohan, Paul Perkins and Martin Dempster in Palliative Medicine

## Supplemental Material

Supplementary_file_4 – Supplemental material for Quality indicators for Palliative Day Services: A modified Delphi study Click here for additional data file. Supplemental material, Supplementary_file_4 for Quality indicators for Palliative Day Services: A modified Delphi study by Noleen K McCorry, Sean O’Connor, Kathleen Leemans, Joanna Coast, Michael Donnelly, Anne Finucane, Louise Jones, W. George Kernohan, Paul Perkins and Martin Dempster in Palliative Medicine
