# Supplementary material for: Quality indicators for Palliative Day Services: A modified Delphi study
Source: Palliat Med. 2018 Nov 19;33(2):197–205. doi: 10.1177/0269216318810601 (PMC6350181; doi:10.1177/0269216318810601)
Supplement: Supplementary_file_1 – Supplemental material for Quality indicators for Palliative Day Services: A modified Delphi study [file Supplementary_file_1.docx]

Supplementary file 1: Original 182 candidate indicators, and panel ratings during round one and two of the RAND/UCLA Appropriateness Method (RAM)

| **#** | **Potential quality indicator descriptions** | **Round 1** | | **Round 2** | | | **Core or supplementary indicator** |
| --- | --- | --- | --- | --- | --- | --- | --- |
|  | **Care Domains** | **Median Appropriateness Score/9 (Range)*** | **Agreement between panel Y/N**† | **Median Appropriateness Score/9**  **(Range)** **‡** | **Median Feasibility Score/9§** | **Rated as necessary by panel**  **Y/N (% yes)¶** |  |
|  | **Physical care and support, assessment and treatment** |  |  |  |  |  |  |
| DQI#01 | Number of patients screened for pain using a validated measure | 8 (5) | Y | 8.5 (3) | 8 | Y (100) | Core |
| DQI#02 | Number of patients with a score >x/10 on a NRS/VAS (average pain on a typical day over the last week) | 3 (8) | N | - | - | - | - |
| DQI#03 | Number of patients with moderate to severe pain | 7 (8) | Y | 7 (5) | 5.5 | Y (55) | Core |
| DQI#04 | For patients who screen positive for pain, the number with any treatment within [x] week[s] | 7 (4) | Y | 8 (3) | 7.5 | N (36) | Supplementary |
| DQI#05 | For patients who screen positive for pain, the number with significant improvement after [x] week[s] | 5.5 (7) | N | 4.5 (4) | 2 | N (45) | - |
| DQI#06 | Number of patients assessed to identify likely cause of pain based on site and radiation (e.g. using a body diagram) | 7 (3) | Y | 8 (3) | 8 | N (27) | Supplementary |
| DQI#07 | Number of patients assessed to identify likely cause of pain based on character (e.g. using a list of descriptors) | 9 (3) | Y | 7.5 (3) | 7.5 | N (27) | Supplementary |
| DQI#08 | Number of patients assessed to identify likely cause of pain based on exacerbating/relieving factors including analgesics | 8.5 (3) | Y | 8 (3) | 8 | N (27) | Supplementary |
| DQI#09 | Number of patients assessed to identify likely cause of pain based on timing and duration | 7.5 (3) | Y | 7 (4) | 7.5 | N (27) | Supplementary |
| DQI#10 | Number of patients assessed to identify likely cause of pain based on effect on function, sleep and mood | 9 (2) | Y | 7.5 (2) | 8 | Y (55) | Core |
| DQI#11 | Documentation of other factors (e.g. emotional, psychological or spiritual) with possible effect on pain perception | 4 (5) | Y | 4.5 (3) | 4.5 | Y (55) | Core |
| DQI#12 | Number of patients screened for SOB using a validated measure | 7 (3) | Y | 8 (4) | 5.5 | N (57) | Core |
| DQI#13 | For patients who screen positive for SOB, the number with any treatment within [x] week[s] | 5 (4) | Y | 3.5 (4) | 7 | N (36) | Supplementary |
| DQI#14 | For patients who screen positive for SOB, the number with significant improvement after [x] week[s] | 3.5 (4) | Y | 7 (3) | 8 | N (64) | Core |
| DQI#15 | Number of patients screened for upper GI (stomach) problems (nausea/vomiting) using a validated measure | 6 (5) | Y | 4 (2) | 5.5 | N (18) | Supplementary |
| DQI#16 | For patients who screen positive for upper GI problems, the number with any treatment within [x] week[s] | 8 (3) | Y | 8 (3) | 8 | N (45) | Supplementary |
| DQI#17 | For patients who screen positive for upper GI problems, the number with significant improvement after [x] week[s] | 7 (5) | N | 4.5 (3) | 3 | N (9) | - |
| DQI#18 | Number of patients screened for lower GI (abdominal) problems (constipation) using a validated measure | 7 (4) | Y | 7.5 (3) | 6 | Y (55) | Core |
| DQI#19 | For patients who screen positive for lower GI problems, the number with any treatment within [x] week[s] | 7 (3) | Y | 5.5 (2) | 7.5 | Y (55) | Core |
| DQI#20 | For patients who screen positive for lower GI problems, the number with significant improvement after [x] week[s] | 9 (2) | Y | 5 (5) | 2 | N (27) | - |
| DQI#21 | Number of patients screened for poor mobility using a validated measure | 6.5 (2) | Y | 3.5 (2) | 7.5 | N (45) | Supplementary |
| DQI#22 | For patients who screen positive for poor mobility, the number with any treatment within [x] week[s] | 6 (8) | N | 5.5 (3) | 1.5 | N (27) | - |
| DQI#23 | For patients who screen positive for poor mobility, the number with significant improvement after [x] week[s] | 3 (5) | N | 4.5 (3) | 2 | N (27) | - |
| DQI#24 | Number of patients screened for tiredness/weakness | 8 (3) | Y | 8 (3) | 8 | Y (55) | Core |
| DQI#25 | For patients who screen positive for tiredness/weakness, the number with any treatment within [x] week[s] | 4 (5) | N | 5.5 (3) | 3 | N (36) | - |
| DQI#26 | For patients who screen positive for tiredness/weakness, the number with significant improvement after [x] week[s] | 5.5 (3) | Y | 3.5 (4) | 5.5 | Y (73) | Core |
| DQI#27 | Number of patients screened for insomnia using a validated measure | 3 (6) | N | - | - | - | - |
| DQI#28 | For patients who screen positive for insomnia, the number with any treatment within [x] week[s] | 3 (4) | Y | - | - | - | - |
| DQI#29 | For patients who screen positive for insomnia, the number with significant improvement after [x] week[s] | 3 (4) | Y | 4 | 5 | Y (55) | Core |
| DQI#30 | Number of patients with an assessment of functional status made using a validated measure reviewed within a [x] month period | 9 (3) | Y | 7 (3) | 5.5 | Y (64) | Core |
| DQI#31 | Number of patients screened for general symptom assessment using a validated measure and reviewed within a [x] month period | 9 (3) | Y | 8 (4) | 8 | Y (91) | Core |
| DQI#32 | Number of patients with a symptom management plan, including goals, agreed in conjunction with the patient and family | 9 (3) | Y | 7.5 (4) | 7.5 | Y (100) | Core |
| DQI#33 | Number of patients with agreed arrangements for regular review of symptoms | 9 (3) | Y | 7 (5) | 7.5 | Y (73) | Core |
| DQI#34 | Number of patients provided with written advice on physical symptom management | 5 (5) | Y | 4 (5) | 2 | N (27) | - |
| DQI#35 | Number of patients who receive an individualized assessment of physical activity levels | 3 (7) | Y | - | - | - | - |
| DQI#36 | Number of patients with an assessment made of satisfaction with overall physical care and support | 8 (2) | Y | 7.5 (3) | 8 | Y (55) | Core |
| DQI#37 | Number of patients satisfied with overall physical care and support assessed using a standardized measure | 3 (8) | Y | - | - | - | - |
|  | **Psychological care and support, assessment and treatment** |  |  |  |  |  |  |
| DQI#38 | Number of patients screened for depression using a validated measure within a [x] month period | 9 (2) | Y | 7.5 (3) | 8 | Y (55) | Core |
| DQI#39 | Number of patients who screen positive for depression, the number with any treatment within [x] week[s] | 5 (6) | N | 6.5 (3) | 4 | Y (55) | Core |
| DQI#40 | Number of patients who screen positive for depression, the number with significant improvement after [x] week[s] | 7 (4) | Y | 8 (4) | 5.5 | Y (55) | Core |
| DQI#41 | Number of patients screened for anxiety using a validated measure within a [x] month period | 9 (1) | Y | 8 (3) | 7.5 | Y (91) | Core |
| DQI#42 | Number of patients who screen positive for anxiety, the number with any treatment within [x] week[s] | 5 (4) | Y | 5.5 (2) | 6 | N (27) | Supplementary |
| DQI#43 | Number of patients who screen positive for anxiety, the number with significant improvement after [x] week[s] | 7 (3) | Y | 7 (3) | 2 | Y (55) | - |
| DQI#44 | Number of patients who receive support when they feel anxious or feel depressed | 3 (7) | N | - | - | - | - |
| DQI#45 | Number of patients diagnosed with depression with a documented treatment plan | 8.5 (2) | Y | 6 (3) | 8.5 | Y (64) | Core |
| DQI#46 | Number of patients diagnosed with depression with response to therapy documented within [x] week[s] | 3 (7) | N | - | - | - | - |
| DQI#47 | Number of patients who receive adequate attention from their caregivers | 1 (8) | N | - | - | - | - |
| DQI#48 | Number of patients are satisfied with the counselling aspects of “politeness” and “being taken seriously” | 1 (7) | N | - | - | - | - |
| DQI#49 | Number of patients who experience respect for their autonomy | 2.5 (8) | N | - | - | - | - |
| DQI#50 | Number of patients who experience respect for their privacy | 2 (4) | Y | - | - | - | - |
| DQI#51 | Number of patients who receive overall quality of life assessment using a validated measure | 9 (3) | Y | 8 (2) | 7.5 | Y (100) | Core |
| DQI#52 | Number of patients who receive condition specific psychological assessment using a validated measure | 1 (8) | N | - | - | - | - |
| DQI#53 | Number of patients referred to / receiving stress management program or intervention | 1.5 (8) | N | - | - | - | - |
| DQI#54 | Number of patients who receive treatment for psychological symptoms in a timely, safe and effective manner | 5 (6) | N | 6 (4) | 6.5 | N (27) | Supplementary |
| DQI#55 | Number of patients with an assessment of cognition performed and results reviewed at least [x] times within [x] months | 9 (2) | Y | 4.5 (3) | 7.5 | Y (64) | Core |
| DQI#56 | Number of patients with neuropsychiatric symptoms receiving or recommended to receive intervention within a [x] month period | 1 (8) | N | - | - | - | - |
|  | **Social care, assessment and management** |  |  |  |  |  |  |
| DQI#57 | Number of patients with a comprehensive social care plan addressing social, practical, and legal needs of patient and caregivers | 1.5 (6) | N | - | - | - | - |
| DQI#58 | Number of patients with equity of access to support for social care needs including counselling services | 1.5 (8) | N | - | - | - | - |
| DQI#59 | Number of patients with a comprehensive interdisciplinary assessment identifying social needs of patients and families | 9 (2) | Y | 8 (3) | 8 | Y (82) | Core |
| DQI#60 | Number of patients with an assigned professional who maintains contact to ensure co-ordinated delivery of services | 6 (5) | N | 5.5 (4) | 4 | N (18) | Supplementary |
|  | **Spiritual and emotional care and support** |  |  |  |  |  |  |
| DQI#61 | Number of patients who indicate that caregivers respect their life stance | 1 (8) | N | - | - | - | - |
| DQI#62 | Number of patients who indicate that they have access to a counsellor for spiritual problems | 3 (7) | N | - | - | - | - |
| DQI#63 | Number of relatives who indicate that the patient had access to a counsellor for spiritual problems | 1 (8) | N | - | - | - | - |
| DQI#64 | Number of relatives who indicate that the patient had accepted her/his approaching death | 1 (8) | N | - | - | - | - |
| DQI#65 | Number of relatives who indicate that there was attention and respect for the spiritual well-being of the patient | 5 (4) | Y | 2 (3) | 1.5 | N (9) | - |
| DQI#66 | Number of relatives who indicate that the patient had access to a counsellor for spiritual problems | 1 (8) | N | - | - | - | - |
| DQI#67 | Number of patients who indicate that they feel that life is worthwhile | 1 (8) | N | - | - | - | - |
| DQI#68 | Number of patients with documentation of discussion of spiritual/religious concerns or that the patient did not want to discuss | 9 (3) | Y | 6 (2) | 8 | Y (82) | Core |
| DQI#69 | Number of patients with a plan based on an assessment of religious, spiritual, and existential concerns using a structured instrument | 9 (4) | Y | 4 (3) | 8 | Y (64) | Core |
| DQI#70 | Number of patients with information about the availability of spiritual care services | 8 (4) | Y | 6 (4) | 8 | Y (91) | Core |
| DQI#71 | Number of patients assessed to identify important spiritual and emotional aspects of care using a validated measure | 6 (4) | Y | 4 (2) | 6.5 | Y (73) | Core |
|  | **Cultural aspects of care** |  |  |  |  |  |  |
| DQI#72 | Number of patients with a non-English native language who have baseline screening performed in their native language | 4.5 (5) | N | 5 (4) | 2 | N (9) | - |
| DQI#73 | Number of patients with access to translators where caregivers and patient / family members do not speak the same language | 8.5 (2) | Y | 7 (3) | 2.5 | Y (64) | - |
| DQI#74 | Number of patients with a cultural assessment including preferences regarding disclosure of information and desire for support measures | 3 (7) | N | - | - | - | - |
| DQI#75 | Number of patients provided with culturally sensitive materials in the patient’s and family’s preferred language | 9 (2) | Y | 5 (6) | 2 | Y (55) | - |
|  | **Generic aspects of care and health promotion** |  |  |  |  |  |  |
| DQI#76 | Extent to which staff assess and manage symptoms and side effects in a timely, safe, and effective manner | 8 (1) | Y | 8 (3) | 7.5 | Y (64) | Core |
| DQI#77 | Number of patients with documented communication between Day Service and General Practitioner | 9 (2) | Y | 8 (4) | 8 | Y (100) | Core |
| DQI#78 | Extent to which patients receive information about the expected course of the illness | 1 (8) | N | - | - | - | - |
| DQI#79 | Extent to which patients receive information about the advantages and disadvantages of various types of treatments | 2.5 (8) | N | - | - | - | - |
| DQI#80 | Extent to which the distinct care needs of patients with different conditions are recognised and addressed | 2 (8) | N | - | - | - | - |
| DQI#81 | Number of patients who receive individual or group patient self-management | 2 (8) | N | - | - | - | - |
| DQI#82 | Number of exercise / rehabilitation sessions attended by patient | 1 (8) | N | - | - | - | - |
| DQI#83 | Number of patients or their caregiver(s) referred for counselling regarding safety concerns within an [x] month period | 1 (8) | N | - | - | - | - |
| DQI#84 | Number of patients provided with education on disease management or referred to additional resources of support | 1 (8) | N | - | - | - | - |
| DQI#85 | Number of patients with assessment and appropriate management of sexual dysfunction | 8 (6) | N | 7 (2) | 8 | Y (52) | Core |
| DQI#86 | Extent to which patients experience respect for their autonomy / privacy | 6 (3) | Y | 4 (4) | 7 | N (36) | Supplementary |
| DQI#87 | Number of patients assessed screened at least once to detect whether they use tobacco regularly | 1 (8) | N | - | - | - | - |
| DQI#88 | Number of patients with an inventory of complaints and problems | 1 (8) | N | - | - | - | - |
| DQI#89 | Number of patients assessed to detect problem drinking by taking a history of alcohol use or using standardized screening questionnaires | 1 (8) | N | - | - | - | - |
| DQI#90 | Extent to which commissioners work to meet needs of patients for complementary therapies where there is evidence to support their use. As a minimum, high quality information should be made available to patients about complementary therapies and services | 9 (2) | Y | 4 (3) | 8 | Y (82) | Core |
| DQI#91 | Extent to which providers ensure practitioners delivering complementary therapies conforms to policies designed to ensure best practice | 9 (2) | Y | 8 (2) | 7.5 | Y (82) | Core |
| DQI#92 | Extent to which commissioners institute mechanisms to ensure patient needs for rehabilitation are met and that services and suitable equipment are available to patients in all care locations | 8.5 (2) | Y | 8.5 (3) | 8.5 | Y (91) | Core |
|  | **Information and communication with patients, carers and family** |  |  |  |  |  |  |
| DQI#93 | Number of patients who indicate that they receive understandable explanations | 9 (1) | Y | 4.5 (6) | 7.5 | N (45) | Supplementary |
| DQI#94 | Number of patients with documentation concerning the desired care and treatment | 8.5 (4) | Y | 5 (3) | 8 | Y (73) | Core |
| DQI#95 | Extent to which patients feel able to communicate their needs in the best possible way, whether verbally or non-verbally | 5 (4) | Y | 3 (3) | 3 | N (45) | - |
| DQI#96 | Extent to which patients feel they are provided with enough information to understand their illness and treatment | 9 (2) | Y | 3 (2) | 7.5 | Y (64) | - |
| DQI#97 | Extent to which patients feel they are given bad news in a sensitive way | 5 (4) | Y | 4.5 (5) | 4 | N (36) | Supplementary |
| DQI#98 | Number of patients with a holistic assessment of palliative care needs of patients and their family caregivers (e.g. SPARC) | 9 (2) | Y | 4 (4) | 8.5 | N (36) | Supplementary |
| DQI#99 | Extent to which, according to the direct relatives, attention was paid to their own psychosocial and spiritual well-being | 1 (8) | N | - | - | - | - |
| DQI#100 | Extent to which the direct relatives felt that they were treated well in all respects by the caregivers | 2 (8) | N | - | - | - | - |
| DQI#101 | Extent to which direct relatives received information that was understandable and unambiguous | 1.5 (8) | N | - | - | - | - |
| DQI#102 | Extent to which direct relatives received information about the advantages and disadvantages of various types of treatment | 1 (8) | N | - | - | - | - |
| DQI#103 | Extent to which patients feel that specialists show an interest in you as a person | 1.5 (8) | N | - | - | - | - |
| DQI#104 | Extent to which family and friends had opportunities to ask questions | 4 (5) | N | 3.5 (4) | 2 | N (36) | - |
| DQI#105 | Extent to which patients feel they have the knowledge and support to make decisions | 8 (2) | Y | 3 (2) | 8.5 | Y (73) | - |
| DQI#106 | Extent to which patients feel that they have opportunities to ask questions | 8 (1) | Y | 5.5 (2) | 4.5 | Y (64) | Core |
| DQI#107 | Number of patients and family/caregivers that understand and are satisfied with provider communication about prognosis | 2 (8) | N | - | - | - | - |
| DQI#108 | Extent to which patients are satisfied with their involvement in decision making | 9 (2) | Y | 8 (3) | 8 | Y (73) | Core |
|  | **Care planning, goal setting and shared decision making with patients, carers and family** |  |  |  |  |  |  |
| DQI#109 | Number of patients with documentation of initial assessment completed within [x] week[s] from referral | 8.5 (4) | Y | 7.5 (3) | 8.5 | Y (64) | Core |
| DQI#110 | Number of patients with regular patient and family care conferences with interdisciplinary team to discuss goals of care and care planning | 2 (8) | N | - | - | - | - |
| DQI#111 | Number of patients with documentation of converted treatment goals into medical orders transferable across settings, for example, through Physician Orders for Life-Sustaining Treatment (POLST) program) | 1 (8) | N | - | - | - | - |
| DQI#112 | Number of patients with advance directives and surrogacy designations available across settings using Internet-based registries or electronic personal health records | 8 (4) | Y | 7 (2) | 8 | Y (55) | Core |
| DQI#113 | Number of patients with documentation of patient/surrogate preferences for goals of care and treatment options at first assessment and at frequent intervals as conditions change | 9 (3) | Y | 8 (2) | 8 | Y (91) | Core |
| DQI#114 | Number of patients with documentation of involvement in decision-making over the past [x] months | 9 (3) | Y | 4.5 (6) | 7.5 | N (45) | Supplementary |
| DQI#115 | Number of patients or caregiver(s) who received 1) comprehensive counselling regarding palliation and symptom management and end of life decisions 2) have advance care plan or surrogate decision maker in the medical record or documentation that patient did not wish or was not able to name a surrogate | 1 (8) | N | - | - | - | - |
| DQI#116 | Number of patients and family/caregivers that understand and are satisfied with their participation in the development of treatment goals | 9 (3) | Y | 5 (4) | 7.5 | Y (55) | Core |
|  | **End of life care and decisions** |  |  |  |  |  |  |
| DQI#117 | Number of patients with documentation of patient and family wishes about the care setting for the site of death, and fulfil patient and family preferences when possible | 1 (8) | N | - | - | - | - |
| DQI#118 | Number of patients with adequate dosage of analgesics and sedatives as appropriate to achieve comfort during the active dying phase, and with addressed concerns and fears about using narcotics and of analgesics hastening death | 3 (6) | N | - | - | - | - |
|  | **Pre and post-bereavement support** |  |  |  |  |  |  |
| DQI#119 | Number of patients with an assessment of psychological reactions of patients and families (including stress, anticipatory grief, and coping) in a regular, ongoing fashion in order to address emotional and functional impairment and loss | 3 (6) | N | - | - | - | - |
| DQI#120 | Number of patients with a grief and bereavement care plan to provide services to patients and families prior to and for at least [x] months after the death of the patient | 3 (6) | N | - | - | - | - |
| DQI#121 | Number of relatives offered counselling for survivors | 3 (6) | N | - | - | - | - |
| DQI#122 | Extent to which the hospice team kept family members informed about the patient’s condition (e.g. always/usually/sometimes/never) | 3 (6) | N | - | - | - | - |
| DQI#123 | Number of relatives who received information from the team about the medicines that were used to manage the patient’s pain | 1.5 (8) | N | - | - | - | - |
| DQI#124 | Number of relatives who received information from the team about what was being done to manage the patient’s trouble with breathing | 3 (8) | N | - | - | - | - |
| DQI#125 | Number of patients where an immediate bereavement plan is activated post-death | 4 (8) | N | 3 (5) | 1 | N (0) | - |
| DQI#126 | Number of relatives who indicate that the patient received support with preparations for saying goodbye | 2.5 (8) | N | - | - | - | - |
|  | **Co-ordination and continuity of care** |  |  |  |  |  |  |
| DQI#127 | Number of patients with a professional caregiver nominated as the responsible ‘key worker‘ who coordinates care | 6 (4) | Y | 2.5 (4) | 2.5 | N (36) | - |
| DQI#128 | Number of patients with a regular review of the care plan based on a comprehensive interdisciplinary assessment of the values, preferences, goals, and needs of the patient and family | 9 (1) | Y | 7.5 (2) | 8 | Y (91) | Core |
| DQI#129 | The extent to which care plans are broadly disseminated to all professionals involved in the patient’s care | 9 (2) | Y | 7 (4) | 8 | Y (64) | Core |
| DQI#130 | The extent to which treatments that are no longer helpful are stopped | 4 (5) | N | 1 (4) | 2 | N (9) | - |
| DQI#131 | The extent to which palliative care services are integrated into the local area health authority and operate at the specific request of the GP and in association with him/her | 1 (8) | N | - | - | - | - |
| DQI#132 | While under the care of hospice, was there always one nurse who was identified as being in charge of the patient’s overall care? (yes/no) | 3 (8) | N | - | - | - | - |
| DQI#133 | Was there any problem with hospice doctors or nurses not knowing enough about the patient’s medical history to provide the best possible care? (yes/no) | 1 (8) | N | - | - | - | - |
| DQI#134 | Time from referral to first contact [calculated as the time in days between the referral date and the date of first contact or episode start date (whichever occurs first)] calculated for all episodes of care and across all settings of care | 7 (3) | Y | 8.5 (4) | 7 | Y (91) | Core |
| DQI#135 | Number of patients with documentation of letter to the referring physician contains the following components: diagnosis; conclusions concerning the care needs of the patient and caregiver(s); medical treatment plan; non-medical treatment plan; advice concerning driving aptitude; care advice for the patent and the caregiver(s) | 9 (5) | N | 7.5 (3) | 8 | Y (100) | Core |
| DQI#136 | Number of patients with a care plan that is revisited with patient and family on a regular basis and following any significant change in health condition | 9 (2) | Y | 8 (4) | 8 | Y (64) | Core |
|  | **Structure and process of care** |  |  |  |  |  |  |
| DQI#137 | Number of patients with documentation of a regular interdisciplinary/multi-professional meeting to discuss management | 9 (2) | Y | 7 (3) | 8 | Y (100) | Core |
| DQI#138 | Number of patients provided with documentation on important complaints that can occur after primary treatment and can be a sign of disease progression | 1 (8) | N | - | - | - | - |
| DQI#139 | Number of patients provided with sufficient time and attention during appointments and after primary treatment has finished | 2 (8) | N | - | - | - | - |
| DQI#140 | Number of patients with a palliative care clinical record containing evidence of: Ethical, legal aspects of care | 1.5 (7) | N | - | - | - | - |
| DQI#141 | Number of patients with a palliative care clinical record containing evidence of: Clinical summary | 9 (2) | Y | 8 (2) | 8 | Y (91) | Core |
| DQI#142 | Number of patients with a palliative care clinical record containing evidence of: Spiritual, religious, existential aspects of care | 8 (2) | Y | 8 (3) | 8 | N (36) | Supplementary |
| DQI#143 | Number of patients with a palliative care clinical record containing evidence of: Physical aspects of care | 8 (2) | Y | 8.5 (4) | 8 | N (45) | Supplementary |
| DQI#144 | Number of patients with a palliative care clinical record containing evidence of: Follow up assessment | 9 (2) | Y | 7.5 (6) | 8 | N (36) | Supplementary |
| DQI#145 | Number of patients with a palliative care clinical record containing evidence of: Psychological and psychiatric aspects of care | 9 (3) | Y | 7.5 (4) | 8 | N (36) | Supplementary |
| DQI#146 | Number of patients with evidence that the care plan was implemented by all providers consistent with goals of care | 1 (7) | Y | - | - | - | - |
|  | **Evidence of effectiveness, outcome assessment and measurement** |  |  |  |  |  |  |
| DQI#147 | Number of patients where a validated tool used to monitor progress (e.g. Edmonton Symptom Assessment System, Edmonton Functional Assessment Tool, Part A of the McGill Quality of Life Index or the Palliative Care Problem Severity Scale) | 7 (3) | N | 8.5 (4) | 8 | Y (64) | Core |
| DQI#148 | Evidence that the palliative care service is involved in research in palliative care (e.g. authorship of publications, research grants) | 1 (8) | Y | - | - | - | - |
| DQI#149 | Number of non-oncological patients receiving palliative care | 4 (5) | Y | 6.5 (7) | 1 | N (9) | - |
| DQI#150 | Number of patients with documentation of adverse events | 9 (2) | Y | 7.5 (4) | 7 | N (45) | Supplementary |
| DQI#151 | Number of patients with evidence of a documented procedure to analyse and follow up adverse events | 9 (2) | N | 7.5 (4) | 7 | N (45) | Supplementary |
| DQI#152 | Number of patients aware of patient complaint procedures | 4 (6) | Y | 3.5 (7) | 4 | N (18) | Supplementary |
| DQI#153 | Number of patients where a patient-reported outcome measures (PROMs) is used that has been validated with relevant populations requiring palliative care and which are sufficiently brief and straightforward and that they allow for proxy reports to be collected when the patient is unable to self-report | 7 (5) | N | 7 (8) | 8 | N (9) | Supplementary |
| DQI#154 | Number of assessment using outcome measures to assess the needs of unpaid caregivers (family and others) alongside the needs of patients | 2.5 (8) | N | - | - | - | - |
| DQI#155 | Evidence of use of change management principles, facilitation and communication to embed outcome measurement into routine clinical practice and evaluate the implementation process to ensure sustained use that penetrates practice within the organisation | 3 (8) | Y | - | - | - | - |
| DQI#156 | Evidence of use of quality improvement systems to sustain routine practice of outcome measurement and institute interoperable electronic systems to ensure integration of measures | 3 (7) | N | - | - | - | - |
| DQI#157 | Evidence of use of established national and international outcome collaborations that work towards benchmarking to establish and improve care standards | 1 (6) | Y | - | - | - | - |
| DQI#158 | Evidence of use of monitoring of palliative care practice through routine collection of outcome data used to establish a minimum dataset of palliative care outcome measures in order to improve and advance care | 6 (4) | Y | 7 (4) | 7 | N (36) | Supplementary |
|  | **Staff training and education, service and professional development** |  |  |  |  |  |  |
| DQI#159 | Number of staff who receive a standardised induction training within [x] month[s] of employment | 9 (2) | Y | 3 (5) | 4 | N (9) | - |
| DQI#160 | All health and social care professionals have standardised learning objectives for continuing basic training in palliative care | 4 (5) | Y | 4 (4) | 1.5 | N (0) | - |
| DQI#161 | Number of staff who professionally deal with loss with access to a program for care for the carers | 1.5 (8) | N | - | - | - | - |
| DQI#162 | Number of staff assessed for satisfaction with working in the team (e.g. Team Climate Inventory) | 7 (6) | Y | 6.5 (4) | 1 | Y (55) | - |
| DQI#163 | Documentation of processes in place to identify the training needs of all workers (registered and unregistered) that take into account the four core common requirements for workforce development (communication skills, assessment and care planning, advance care planning, and symptom management) as they apply to end of life care | 9 (3) | Y | 4.5 (4) | 8 | N (27) | Supplementary |
| DQI#164 | Number of staff with access to curricula for training as part of continuing professional education including palliative care for patients with illness other than cancer | 7 (6) | Y | 7.5 (3) | 7.5 | Y (64) | Core |
|  | **Access to services and service environment** |  |  |  |  |  |  |
| DQI#165 | Number of patients who have access to diagnostic investigations (e.g. x-rays, blood samples] regardless of the setting | 1 (8) | Y | - | - | - | - |
| DQI#166 | Number of patients receiving palliative care provided with transportation to the service | 1 (8) | Y | - | - | - | - |
| DQI#167 | Number of patients experiencing a crisis where the following is arranged within [x] hours: admission | 1.5 (7) | Y | - | - | - | - |
| DQI#168 | Number of patients receiving the following treatments as needed 24 hours a day, [x] days a week: opioids and other controlled drugs | 8 (2) | N | 3.5 (7) | 4 | N (18) | Supplementary |
| DQI#169 | There is a dedicated room where multidisciplinary team meetings within one setting takes place | 3 (7) | N | - | - | - | - |
| DQI#170 | Relevant services and care providers should ensure equal access to available day services based on need through appropriate referrals | 9 (2) | N | 3 (5) | 9 | N (45) | - |
| DQI#171 | All service users should have equity of access to all day services and support that is available | 3 (6) | N | - | - | - | - |
| DQI#172 | If a confidential discussion has to take place then it should take place in private | 1.5 (8) | Y | - | - | - | - |
| DQI#173 | The setting of care should meet the preferences, needs, and circumstances of the patient and family to the extent possible | 8.5 (4) | N | 4.5 (4) | 4.5 | Y (82) | Core |
| DQI#174 | In rural areas where accessing specialized care is difficult, organizations should institute telehealth and telemedicine communications | 4 (6) | Y | 3.5 (4) | 3 | N (0) | - |
|  | **Promotion of effective external engagement** |  |  |  |  |  |  |
| DQI#175 | Number of patients provided with access to an up to date directory of local caregivers and organisations | 1 (8) | N | - | - | - | - |
| DQI#176 | Number of patients provided with access to dedicated information about the palliative care service: A website | 8 (6) | Y | 7 (3) | 5 | Y (82) | Core |
| DQI#177 | Number of patients provided with access to dedicated information about the palliative care service: Leaflets or brochures | 9 (7) | Y | 6.5 (3) | 7 | Y (91) | Core |
| DQI#178 | Develop healthcare and community collaborations to promote advance care planning and the completion of advance directives for all individuals(for example, the Respecting Choices and Community Conversations on Compassionate Care programs) | 8.5 (4) | Y | 8.5 (3) | 7 | Y (82) | Core |
| DQI#179 | Processes will be in place to manage ethical aspects involving discordant patient, family, and caregiver goals and to handle disputes and uncertainties regarding a patient’s previously stated preferences and current family or proxy decisions. | 8 (7) | Y | 5 (5) | 9 | Y (55) | Core |
|  | **Societal, ethical and legal aspects of care** |  |  |  |  |  |  |
| DQI#180 | Number of patients with documentation of informed consent before therapy | 9 (4) | Y | 8 (4) | 9 | Y (91) | Core |
| DQI#181 | Annual reports are produced reporting the activities and characteristics of the service such as team composition, staff composition, resources, referring physicians, patient characteristics | 4 (3) | Y | 1.5 (8) | 3 | N (0) | - |
| DQI#182 | All patients living in psychosocial circumstances presenting a high-risk for their health should be identified as soon as possible | 3 (2) | Y | - | - | - | - |

*Median Appropriateness Score /9 (Range): Appropriateness ratings of 1-3 were categorized as inappropriate, ratings of 4-6 were classified as uncertain and ratings 7-9 were classified as appropriate.

†Agreement between panel Y/N: With a panel size of 16, level of agreement was based on a maximum of four panel members rating a quality indicator outside a three-point region around the median value; with disagreement defined as five or more panel members rating an indicator at the extreme ends of the scale as either inappropriate (1-3) or appropriate (7-9).

§Median Feasibility Score /9: Feasibility was assessed using the same 9 point scale as appropriateness, and level of agreement was based on a maximum of three panel members rating a quality indicator outside a three-point region around the median value; with disagreement defined as four or more panel members rating an indicator at the extreme ends of the scale as either inappropriate (1-3) or appropriate (7-9). Level of agreement data for feasibility are not shown

¶Rated as necessary by panel members Y/N (% yes).
