# Supplementary material for: Quality indicators for Palliative Day Services: A modified Delphi study
Source: Palliat Med. 2018 Nov 19;33(2):197–205. doi: 10.1177/0269216318810601 (PMC6350181; doi:10.1177/0269216318810601)
Supplement: Supplementary_file_2 – Supplemental material for Quality indicators for Palliative Day Services: A modified Delphi study [file Supplementary_file_2.docx]

Supplementary file 2: Derivation of final indicator set (n=30) from original candidate indicators, including indicators combined during RAM round 3

|  | **Final indicator description following phase three of RAND/UCLA Appropriateness Method** | **Original (combined) candidate indicators** |
| --- | --- | --- |
|  | **A. Physical care and support, assessment and treatment** |  |
| A1 | Proportion of service users with assessment of pain severity at screening using a valid measure | #01 |
| A2 | Proportion of service users with moderate or severe pain assessed to explore possible causes of pain | #03,#10,#11 |
| A3 | Proportion of service users with assessment of breathlessness at screening using a valid measure | #12 |
| A4 | Proportion of service users with assessment of fatigue at screening using a valid measure | #24 |
| A5 | Proportion of service users with assessment of functional status to identify daily activity limitations completed before a multidisciplinary care plan | #30 |
|  | **B. Psychological care and support, assessment and treatment** |  |
| B6 | Proportion of service users screened for depression at screening using a valid measure | #38 |
| B7 | Proportion of service users screened for anxiety at screening using a valid measure | #41 |
| B8 | Proportion of service users with assessment of cognitive functioning | #55 |
|  | **C. Spiritual and emotional care and support** |  |
| C9 | Proportion of service users with documentation of a spiritual aspects of care discussion or assessment completed before a multidisciplinary care plan | #68;#71 |
|  | **D. Information and Communication with Service Users** |  |
| D10 | Proportion of service users who report that they are provided with sufficient, appropriately tailored information or advice on their condition and on intervention options to support decisions on agreed care planning | #177,#179 |
|  | **E. Co-ordination and continuity of care** |  |
| E11 | Proportion of service users with a comprehensive needs assessment completed before a multidisciplinary care plan to identify main symptoms and concerns and their effect | #59,#85,#109 |
| E12 | Service has a written care pathway for assessment and management of moderate or severe pain including appropriate onward referral routes | #10,#11 |
| E13 | Proportion of service users with documentation of re-assessment at regular review in line with time points agreed in the multidisciplinary care plan | #128,#136,#137,#147 |
| E14 | Service has a written standard operating procedure defining timeframes for time to initial contact, completion of needs assessment and multidisciplinary care plan | #33, #94 |
| E15 | Proportion of service users with documentation of appropriate intervention in line with the agreed, multidisciplinary care plan | #39, #70, #76 |
| E16 | Proportion of service users with documented communication between the service and the General Practitioner providing information on care needs and the agreed care plans | #77,#135 |
| E17 | Proportion of service users with a care plan available as specified by the service’s written standard operating procedure for development and usage of multidisciplinary care plans | #129,#141 |
| E18 | Proportion of service users with documented evidence of being offered the opportunity for completion of advance care planning | #178 |
| E19 | Proportion of service users with quality of life assessed using a valid measure at screening and at regular review in line with time points agreed in the multidisciplinary care plan | #51 |
|  | **F. Care planning, goal setting and shared decision making with service users** |  |
| F20 | Service has a written standard operating procedure for development and usage of multidisciplinary care plans | #109 |
| F21 | Proportion of service users with documentation of main care goals in the multidisciplinary care plan | #32,#33,#45,#69,#94,#113 |
|  | **G. Evidence of effectiveness, outcome assessment and measurement** |  |
| G22 | Service has a written policy for reviewing and updating standard operating procedures and care pathways |  |
| G23 | Proportion of service users re-assessed at regular review who report that main care goals are met in line with the multidisciplinary care plan | #14,#26,#29,#40 |
| G24  G25 | Proportion of service users with assessment of satisfaction with overall care and support performed using a valid measure  Proportion of service users with assessment of satisfaction with involvement in shared decision making | #37  #106, #108, #116 |
|  | **H. Staff training and education, service and professional development** |  |
| H26 | Extent to which staff have access to training around core components of care as part of continuing education and personal development | #164 |
|  | **I. Access to services and service environment** |  |
| I27 | Proportion of service users with a record of time in days from referral date to first attendance date offered by service | #134 |
| I28 | The service provides suitable equipment and settings to deliver care | #92,#173 |
| I29 | Service has a written policy for defining standards for equipment and settings which are available for delivery of care | #92 |
|  | **J. Societal, ethical and legal aspects of care** |  |
| J30 | Number of service users with correctly completed documentation of informed consent to treatment or medical intervention | #180 |
