# Supplementary material for: Quality indicators for Palliative Day Services: A modified Delphi study
Source: Palliat Med. 2018 Nov 19;33(2):197–205. doi: 10.1177/0269216318810601 (PMC6350181; doi:10.1177/0269216318810601)
Supplement: Supplementary_file_3 – Supplemental material for Quality indicators for Palliative Day Services: A modified Delphi study [file Supplementary_file_3.docx]

Supplementary file 3: Extract from the QualPalUK toolkit, showing data extraction required for quality indicator A1 (Proportion of service users with assessment of pain severity at screening using a valid measure)

| **LOGO** | **PALLIATIVE DAY SERVICE QUALITY INDICATOR SET:**  **DATA ABSTRACTION FORM A [PATIENT LEVEL INDICATORS]** | **ABSTRACTOR:** | **DATE:** | **SITE:** |
| --- | --- | --- | --- | --- |

| **DATA SAMPLE & REFERENCE PERIOD:** | | | ***e.g. 15 consecutive service users discharged in period (INSERT DATES)*** | | |
| --- | --- | --- | --- | --- | --- |
| **QI** | **DENOMINATOR** |  | | | **Please use table to abstract the required information from each clinical record**   \| **#** \| **Assessment**  **documented** \| **Assessment during screening** \| **Valid Measure**  **used** \| **State measure used** \| **Comments** \| \| --- \| --- \| --- \| --- \| --- \| --- \| \| **1** \|  \|  \|  \|  \|  \| \| **2** \|  \|  \|  \|  \|  \| \| **3** \|  \|  \|  \|  \|  \| \| **4** \|  \|  \|  \|  \|  \| \| **5** \|  \|  \|  \|  \|  \| \| **6** \|  \|  \|  \|  \|  \| \| **7** \|  \|  \|  \|  \|  \| \| **8** \|  \|  \|  \|  \|  \| \| **9** \|  \|  \|  \|  \|  \| \| **10** \|  \|  \|  \|  \|  \| \| **11** \|  \|  \|  \|  \|  \| \| **12** \|  \|  \|  \|  \|  \| \| **13** \|  \|  \|  \|  \|  \| \| **14** \|  \|  \|  \|  \|  \| \| **15** \|  \|  \|  \|  \|  \| |
| **A1** | **Number of sample attending day hospice during reference period** | | | ***N=15*** |  |
|  | **NUMERATOR** |  | | |  |
|  | **Number of service users with assessment of pain severity at screening using a valid measure** | | | **-** |  |
|  | **Required criteria:**   - **Pain severity assessment documented in notes** - **Pain severity assessed completed during screening [within 1-3 visits]** - **Pain severity assessed using a valid measure** | | | |  |
|  | **Key terms/definitions:**  • **Service users: Patients attending day hospice**  **• Pain severity: Unidimensional assessment of current pain level using a valid measure and accepted descriptors of pain severity or intensity (e.g., Pain Visual Analogue Scale (VAS), Pain Numerical Rating scale (NRS) or Pain Verbal Rating Scale (VRS)****  **• Screening: Processes of assessment undertaken during the early stages (within 1-3 visits) of attendance at day service, at triage, or at the beginning of a new episode or phase of care**  **• Valid measure: The measure is appropriate and has acceptable validity and reliability when used according to specific instructions**  **** Observational or other non, self-reported measures are not included in assessment of this indicator** | | | |  |
