# Supplementary material for: Quality indicators for Palliative Day Services: A modified Delphi study
Source: Palliat Med. 2018 Nov 19;33(2):197–205. doi: 10.1177/0269216318810601 (PMC6350181; doi:10.1177/0269216318810601)
Supplement: Supplementary_file_4 – Supplemental material for Quality indicators for Palliative Day Services: A modified Delphi study [file Supplementary_file_4.docx]

Supplementary file 4: Practice test results: combined (across 5 settings) mean performance on each indicator.

| **QI** | **Patient and staff level indicators*** | **Mean (%)** | **SD** | **Range** |
| --- | --- | --- | --- | --- |
| **A1** | Pain severity assessed at screening using a valid measure | 76.8 | 10.2 | 67-93 |
| **A2** | If moderate or severe pain present, patient assessed to explore possible causes of pain | 63.8 | 15.1 | 50-87 |
| **A3** | Breathlessness assessed at screening using a valid measure | 73.2 | 13.8 | 55-86 |
| **A4** | Fatigue assessed at screening using a valid measure | 68.8 | 16.6 | 47-93 |
| **A5** | Functional status assessed to identify daily activity limitations prior to formulating care plan | 64.8 | 17.3 | 46-93 |
| **B6** | Depression assessed at screening using a valid measure | 49.4 | 21.3 | 27-75 |
| **B7** | Anxiety assessed at screening using a valid measure | 45.6 | 9.2 | 34-56 |
| **B8** | Cognitive function assessed | 56.0 | 17.1 | 32-78 |
| **C9** | Spiritual aspects of care discussion or assessment completed before care plan | 59.8 | 21.6 | 26-85 |
| **D10** | Patient provided with sufficient information or advice to support decisions on care planning | 16.4 | 4.5 | 10-21 |
| **E11** | Comprehensive needs assessment completed before care plan | 42.2 | 29.2 | 0-78 |
| **E13** | Re-assessment made at regular review in line with time-points agreed in care plan | 71.8 | 28.8 | 23-93 |
| **E15** | Appropriate intervention documented in line with the agreed care plan | 54.6 | 25.5 | 28-93 |
| **E16** | Communication between the service and general practitioner documented | 89.2 | 17.6 | 58-100 |
| **E17** | Care plan available as specified by standard operating procedure | 15.0 | 22.4 | 0-54 |
| **E18** | Opportunity offered for completion of advance care planning | 56.0 | 20.3 | 37-90 |
| **E19** | Quality of life assessed using a valid measure | 11.0 | 15.6 | 0-38 |
| **F21** | Care goals documented in care plan | 65.2 | 20.9 | 32-86 |
| **G23** | Care goals met at regular review in line with care plan | 35.0 | 33.4 | 12-93 |
| **G24** | Assessment of satisfaction with overall care and support using a valid measure | 43.4 | 27.9 | 0-78 |
| **G25** | Assessment of satisfaction with involvement in decision making | 17.6 | 36.1 | 0-82 |
| **I27** | Time in days from referral date to first attendance date offered | 95.8 | 6.2 | 86-100 |
| **J30** | Documentation of informed consent to treatment or medical intervention correctly completed | 93.4 | 11.2 | 74-100 |
| **H26** | Staff with access to training around core components of care | 100.0 | 0.0 | 100-100 |

| **QI** | **Service level indicators*** | **% of services meeting the QI** |
| --- | --- | --- |
| **E12** | Care pathway for assessment and management of pain including onward referral routes | 40% (2/5) |
| **E14** | Written standard operating procedure defining timeframes | 20% (1/5) |
| **F20** | Written standard operating procedure for development and usage of care plans | 40% (2/5) |
| **G22** | Written policy for reviewing and updating standard operating procedures and care pathways | 40% (2/5) |
| **I28** | The service provides suitable equipment and settings to deliver care | 60% (3/5) |
| **I29** | Written policy for defining standards for equipment and settings available for delivery of care | 60% (3/5) |

*Abbreviated indicator names
